# Sensor fusion and downscaled climate projections reveal climate refugia in the California Channel Islands

**DOI:** 10.1038/s41598-026-56345-4

**Published:** 2026-06-08

**Authors:** Erica M. Gallerani, Stacey Ostermann-Kelm, Cameron B. Williams, G. Andrew Fricker, Kyle C. Cavanaugh, Thomas W. Gillespie

**Affiliations:** 1 Department of Geography, University of California Los Angeles, Los Angeles, CA USA; 2 Mediterranean Coast Network, NPS Inventory & Monitoring Program, 401 West Hillcrest Dr., Thousand Oaks, CA 91360 USA; 3 Division of Natural Resources, Channel Islands National Park, Ventura, CA 93001 USA; 4 Social Sciences Department, California Polytechnic University, San Luis Obispo, CA USA

**Keywords:** Climate sciences, Ecology, Ecology, Environmental sciences

## Abstract

Conservation actions such as habitat restoration and translocation require spatially informed, quantitative decision-making. We modeled habitat suitability for nine priority landbird species of the Channel Islands to understand habitat preferences, inform conservation planning and assess future climate impacts. In the absence of recent airborne lidar, we derived vegetation structure from spaceborne lidar, radar and optical remote sensing data combined with downscaled climate observations to train machine-learning models. Models performed well (average AUC = 0.84) under spatial cross-validation. Incorporating citizen science data improved Boyce Index performance for eight of the nine species, though AUC declined for 6 out of 9 species, likely reflecting spatial bias in opportunistic records. A radar-derived vegetation index was consistently influential, while maximum temperature strongly affected species restricted to Santa Cruz Island. Most species are projected to lose suitable habitat under two near-future (2040–2069) climate scenarios, except Grasshopper sparrow, Island scrub jay and Rufous-crowned sparrow. We identified climate change refugia to inform spatial conservation priorities.

**Supplementary Information:**

The online version contains supplementary material available at 10.1038/s41598-026-56345-4.

## Introduction

Experts estimate that about 30% (uncertainty range: 16–50%) of global species have been threatened or driven to extinction since the year 1500, likely decreasing ecosystem functioning and services^1^. Habitat loss and degradation are considered major drivers of this biodiversity loss, with impacts of these threats likely to increase under a changing climate^2^. Climate adaptation and mitigation strategies such as habitat restoration, identifying climate refugia, and translocation are essential to reverse species declines and prevent extinctions^3,4^. Restoration of degraded habitats can contribute to viable and persistent populations of threatened species. However, these efforts are often limited by logistical and economic challenges. Climate change refugia are areas protected from contemporary climate change that allow species to persist under future climate scenarios, making their identification crucial for conservation^5,6^. Previous research has found that key challenges affecting the effectiveness of species recovery plans include inadequate integration of scientific knowledge and insufficient data for identifying critical habitat^7^. A combination of remote sensing and machine learning in habitat suitability modeling could be a low-cost, time-efficient, and accurate tool for filling these gaps in conservation planning and increase the likelihood of intervention success. The incorporation of climate data into these frameworks allows these tools to be forward-looking, which is essential for understanding the effects of climate change on biodiversity and conservation planning.

### Forest structure

Forest structure has been shown as an essential determining factor of habitat suitability across taxa but in particular with avian species^8^. Synthetic aperture radar (SAR) and light detection and ranging (lidar) are two forms of active remote sensing which allow vegetation structure to be measured at greater spatial scales and resolutions than ever before^9^. New advances in spaceborne lidar projects such as the NASA Global Ecosystem Dynamics Investigation (GEDI) attempt to fill the gaps of airborne laser scanning coverage globally. GEDI uses ~ 25 m diameter footprint full-waveform lidar data to measure vegetation structure and began collecting data in April of 2019 from the International Space Station^10^. Sensor fusion usually involves the use of machine learning to build relationships between data from two different spaceborne or airborne sensors. Incorporating freely available public data can allow for the assessment of vegetation structure over time and provides coverage in areas previously data poor due to high costs of lidar data acquisition^11^. These data fusion methods are essential for areas that have experienced drastic changes in vegetation structure due to natural disasters and human or animal intervention.

### Study area

**Fig. 1:**
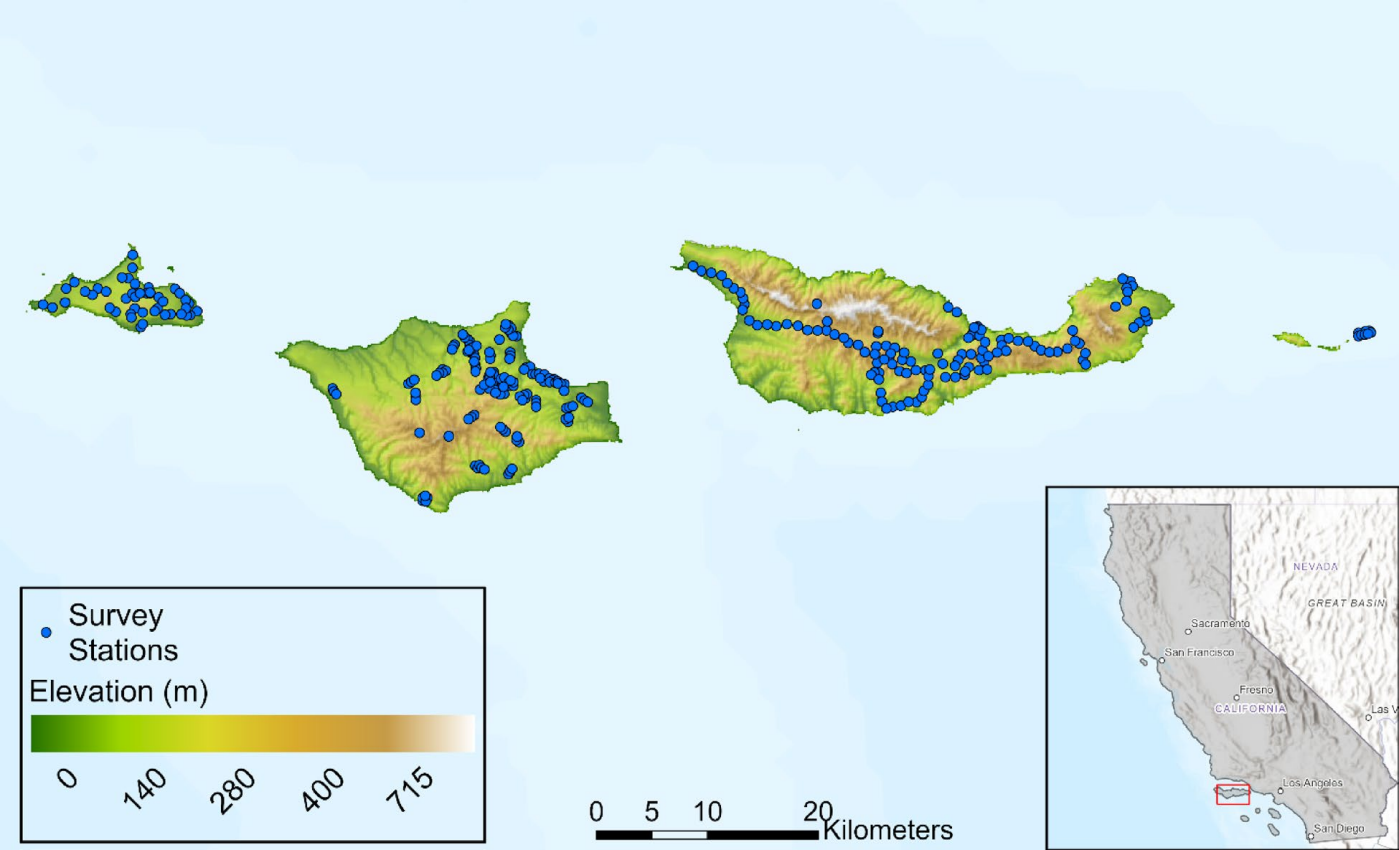
A map of the northernmost of California Channel Islands with San Miguel to the West, then Santa Rosa, Santa Cruz and Anacapa. The blue dots represent regular NPS landbird monitoring survey stations and the colors on the island represent elevation values ranging from 0 m above sea level to 716 m above sea level on Santa Cruz Island. This map was generated using ArcGIS Pro Software Version 3.4.0^17^.

The Channel Islands, one of Californias most biodiverse regions, are an archipelago separated from the coast of southern California by the Santa Barbara Channel (California Department of Fish and Wildlife 2021). Channel Islands National Park (CHIS) encompasses five of the eight islands in the archipelago: Anacapa, Santa Cruz, Santa Rosa, San Miguel and Santa Barbara. These islands range in size from the small island of Anacapa (2.8 km^2^) and Santa Barbara (2.6 km^2^) to the largest islands of Santa Rosa (215 km^2^) and Santa Cruz (249 km^2^) with San Miguel being 37.7 km^2^ (Fig. 1). Sheep, cattle, pigs, burros, horses, deer, elk, rats, cats and rabbits have all been eradicated from the islands with some eradication occurring as recently as 2014^12,13^. Given these eradications, the park has experienced drastic changes in vegetation cover and type, moving away from invasive grasslands and towards native chaparral and scrub shrubland^14–16^. The most recently available ALS data for the park was collected in October and November of 2010. This makes the Channel Islands National Park an ideal use case for sensor fusion to provide the most up-to-date vegetation structure data.

### Climate

The Channel Islands experience a Mediterranean dry summer subtropical climate with temperatures largely controlled by the sea and variable inter-island winter precipitation averaging between 150 and 360 mm^18^. The islands are home to a high richness of rare bird species, the threats to which will likely be exacerbated by climate change^19^. Island populations with limited dispersal ability are particularly vulnerable to shifts in climate^20^. Changes in precipitation in the Channel Islands will affect wildfire risk and food availability^21^. Changes in temperature could increase the prevalence of mosquito-vectored disease such as West Nile Virus and other pests or pathogens which threaten habitat for native birds such as the goldspotted oak borer (*Agrilus auroguttatus*)^22^. These threats make accurate predictions of future climate for these islands essential for avian conservation planning. In this study we focus on nine species that are either endemic species or subspecies, declining on the islands, and/or threatened as determined by stakeholders at NPS, the Smithsonian Institute, Western Foundation of Vertebrate Zoology, and the U.S. Geological Survey (Supplemental Material Table 1). These species are Chipping Sparrow (*Spizella passerina*), Grasshopper Sparrow (*Ammodramus savannarum*), Hutton’s Vireo (*Vireo huttoni*), Island Scrub Jay (*Aphelocoma insularis*), Loggerhead Shrike (*Lanius ludovicianus anthonyi*), Orange-crowned Warbler (*Leiothlypis celata sordida*), Rufous-crowned Sparrow (*Aimphila ruficeps obscura*), Song Sparrow (*Melospiza melodia*), and Western Meadowlark (*Sturnella neglecta*). Notably, ongoing vegetation change on the islands, particularly the transition from non-native grasslands toward native chaparral and scrub shrubland following herbivore removal, is considered a potential driver of decline for grassland-associated species. Grasshopper Sparrow, Western Meadowlark, and the Loggerhead Shrike subspecies were identified as species of particular concern by NPS staff given this trajectory of habitat loss, and this context informed their inclusion in the study.

## Research objectives

This research has three primary objectives. First, we examine the utility of active remote sensing (GEDI, Sentinel-1) for quantifying vegetation structure on the Channel Islands and compare results to field methods. Second, we create species distribution models for nine priority land birds over the three largest islands to identify environmental metrics associated with species current habitat suitability and compare results from National Park Service monitoring and citizen science datasets. Third, we project these models of habitat quality into two near-future (2040–2069) climate scenarios using downscaled projections from an ensemble of eight CMIP5 models to identify species vulnerable to climate change and identify potential climate change refugia.

## Results and discussion

### GEDI based random forest model performance and correction

**Fig. 2:**
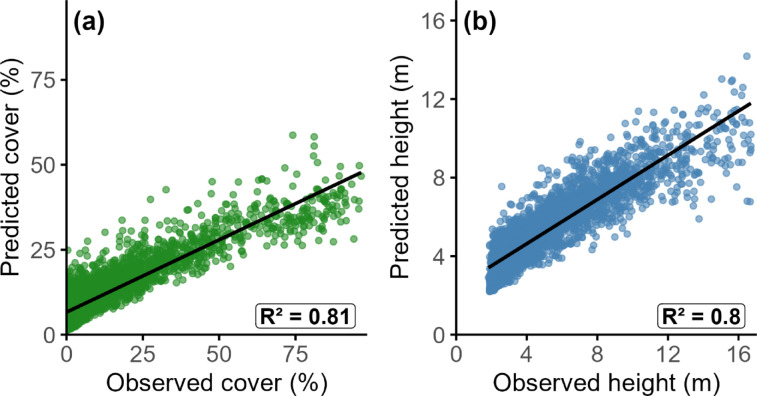
Observed GEDI measurements of percent vegetation cover (green) on the left and vegetation height (m) (blue) on the right along the x-axes with percent vegetation cover and vegetation height (m) predicted by the random forest model on the y-axis.

After filtering the GEDI height points there were a total of 17,475 points for training and testing of each tree in the random forest model, with the full set of GEDI points (25,474) used for training and testing the vegetation percent cover model. The estimated error for the height model was 2.71 m with highest relative variable importances assigned to the Landsat short wave infrared band 1 reflectance, green band reflectance, elevation and Landsat near infrared band reflectance in that order. The estimated error for the cover model was 0.167 with the highest relative variable importance assigned to the Landsat shortwave infrared band 1 reflectance, red band reflectance, green band reflectance, and near infrared reflectance. Variable importance in both models align well with common methods for studying vegetation with remote sensing. Importantly SWIR1 has been found to be highly sensitive to changes in canopy leaf area index, mean leaf angle, and vegetation water content^23^. Elevation has a large impact on the vegetation height model likely because of the relationship between elevation and available moisture as fog inundation follows an elevation gradient on the islands^24^. Strong correlations ($\:\rho\:\ge\:$ 0.8) were found between predicted 30 m percent cover and vegetation height and the observed GEDI data. These results indicate that the random forest models built on Landsat and DEM data performed well in predicting GEDI measurements of vegetation height and cover in CHIS (Fig. 2).

The NPS vegetation survey data from 2021 to 2024 resulted in 815 ground measurements used as validation for the height random forest model. The RMSE of our random forest model compared to NPS ground measurements was 4.3 m, highlighting the need for ground validation of vegetation height models especially over areas covered by lower vegetation heights. Our results corroborate findings from Lang et al.,^25^ which found overestimation in vegetation heights < 5 m in their global model built using deep learning, GEDI height data, and Sentinel 1 imagery, with an RMSE of 5.5 m over the Channel Islands. After the application of a simple GAM model correction, built on recent ground validation data from Santa Rosa, San Miguel and eastern Santa Cruz islands, our RMSE was reduced to 1.47 m with a bias of 0.26 m. Additionally, when using independent data measured on Santa Cruz Island, our corrected vegetation height model had a similar RMSE of 1.50 m with a negative bias of -0.63 m (Supplemental Material Fig. 1). This suggests that our corrected height model overestimates height for the islands with shorter vegetation and underestimates height for the tallest vegetation (Supplemental Material Fig. 1).

Previous studies of vegetation trends on the California Channel Islands have reported overall stability in NDVI between 2000 and 2016, along with increased cover of native vegetation types such as woodlands, chaparral, and scrub shrubland between 1986 and 2011^14,16^. Our percent vegetation cover and vegetation height models support the continuation of this trend, showing increased chaparral cover on San Miguel and Santa Rosa Islands and expanded scrub shrubland at higher elevations on Santa Rosa Island. These changes are reflected in increases in both vegetation height and cover relative to 2010 airborne lidar data, and are consistent with vegetation mapping and long-term monitoring that document increases in native shrub cover, density and recruitment^15,16^. The observed increases are likely a result of the removal of introduced herbivore species, which has facilitated the recovery of taller, denser native vegetation in areas previously dominated by non-native grasslands^26,27^.

Decreases that are seen between the ALS 2010 data and our vegetation height model are likely driven in part by underestimation of the tallest vegetation in our height model, particularly after the GAM correction which missed ground validation data from Western Santa Cruz Island. However, McIntyre et al.^28^ show that California forests (including areas similar to Santa Cruz Island woodlands) are experiencing reductions in height and increases in density mostly driven by increases in climatic water deficit (CWD). They also find shifts away from taller pines towards shorter oaks given the water limitations facing these ecosystems. A recent NPS study documented a declining trend in the odds of native tree cover on Santa Cruz Island from 2000 to 2018^15^. It is possible that some signal of this climate-driven trend in changing vegetation structure is being captured by the lower vegetation height in the woodlands in Santa Cruz Island.

### Model performance and evaluation

Most species model parameters were limited to linear and quadratic feature classes with a regularization multiplier of 1. These simple models suggest high transferability for projecting to future scenarios and follow recommendations for presence-only modeling^29^. The endemic subspecies of Rufous-crowned Sparrow, had the most complex model with linear, quadratic and hinge feature types and a regularization multiplier of 5. The average AUC of the nine species models was 0.84 using NPS data and 0.83 using NPS and citizen science (GBIF) data. The average Boyce index of the nine species models was 0.95 using NPS data and 0.97 using NPS and citizen science (GBIF) data. Species models with the highest AUC scores ($\:\ge\:$ 0.85) are either restricted to one island (Island Scrub Jay and Rufous-crowned Sparrow subspecies) or have specialized habitat use as is the case for the Grasshopper Sparrow and Hutton’s Vireo. Generalist species such as the Song Sparrow and Orange-crowned Warbler subspecies had the lowest performance (AUC = 0.775) as they can occupy a wide range of habitats and are found at nearly every survey station in the three islands studied. A systematic study of 64 bird species in Spain found that when controlling for the positive effect of prevalence, the better predicted species had high environmental specialization^30^, which supports similar conclusions amongst other taxa^31^. However, the Boyce Index showed an opposite pattern for performance, with Song Sparrow and the Orange-crowned Warbler subspecies being the best performing models and Grasshopper Sparrow having amongst the worst performance.

Additionally, we found that the source of the species data also influenced performance. Incorporating research-grade citizen science data sourced from GBIF produced mixed results depending on the performance metric used. When assessing AUC, adding GBIF data decreased performance for 6 of 9 species, suggesting that spatial bias in opportunistic observation can reduce discriminatory power in spatially cross-validated models (Table 1). However, the Boyce Index improved for 8 of 9 species when GBIF data were included, suggesting that supplemental citizen science records improved model calibration even where discrimination declined. Habitat suitability modeling literature points to how sampling bias can lead to artificially high-performance evaluations, and in an area where thinning would lead to very small sample sizes, using only citizen science data for modeling is unfeasible^32^. Citizen science data in CHIS has a strong spatial bias towards accessible parts of the park which can have significant negative impacts on model accuracy^33^. According to guidelines laid out by Araujo et al., (2019), the gold standard for species data in a distribution model is repeated systematic surveying with sufficient and well-designed sampling as is the case for the NPS landbird monitoring species data. We therefore use NPS only models throughout the remainder of the study. Most species had a standard deviation of ~ 0.01 between the sub models suggesting a low level of uncertainty in the average predicted probability of presence results for current conditions for each species (Table 1).

**Table 1:** Average AUC and Boyce Index amongst the Maxent sub-models built on unique training data points from 2014-present from NPS surveys and that data combined with citizen science data (GBIF).

Species name	Models trained with NPS Only			Models trained with NPS and GBIF Data		
	AUC (SD)	Boyce Index (SD)	*N*	AUC (SD)	Boyce Index (SD)	*N*
Chipping sparrow	0.809 (0.008)	0.973 (0.017)	188	0.805 (0.014)	**0.978 (0.011)**	240
Grasshopper sparrow	0.85 (0.011)	0.855 (0.035)	33	0.829 **(0.009)**	**0.936 (0.028)**	56
Hutton’s vireo	0.878 (0.009)	0.961 (0.009)	116	0.871 (0.009)	**0.969** (0.015)	167
Island scrub jay	0.904 (0.008)	0.931 (0.021)	97	**0.913** (0.009)	**0.967 (0.019)**	201
Loggerhead shrike	0.841 (0.019)	0.922 (0.020)	79	0.832 (0.02)	**0.943** (0.027)	127
Orange-crowned warbler	0.775 (0.008)	0.984 (0.008)	260	**0.783** (0.015)	0.981 (0.012)	355
Rufous-crowned sparrow	0.883 (0.01)	0.958 (0.008)	75	**0.891 (0.009)**	0.958 (0.047)	124
Song sparrow	0.775 (0.009)	0.983 (0.011)	249	0.768 (0.014)	**0.988 (0.004)**	369
Western meadowlark	0.814 (0.013)	0.972 (0.027)	189	0.778 (0.015)	**0.984 (0.012)**	277

SD is the standard deviation between the sub-models as a measure of uncertainty. N represents the unique number of training points available to each sub-model. Increases in performance or reduction in uncertainty between NPS and NPS and GBIF models are in bold.

### Variable importance and response curves

**Fig. 3:**
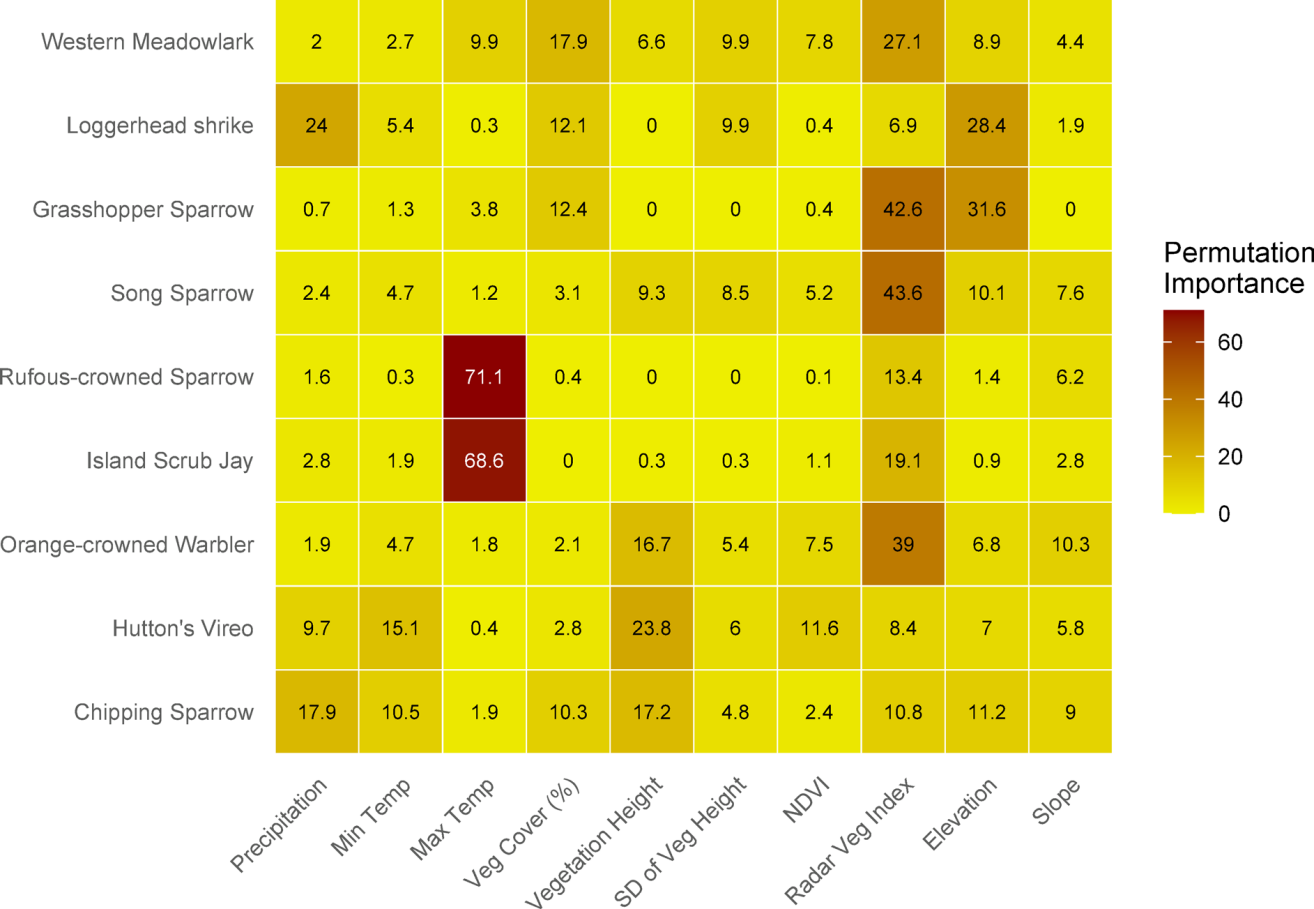
The permutation importance (%) for each variable on the x-axis per species on the y-axis. Yellow colors represent lower permutation importance with higher permutation importance values represented in red.

There is a degree of collinearity amongst predictor variables, making variable importance interpretation complicated. Cover, vegetation height, standard deviation of height and terrain slope all have variable inflation factors greater than 5 (Supplemental Methods). All vegetation structure metrics produced through GEDI and Landsat random forest models are highly correlated to each other (> 0.70). Vegetation height, standard deviation of height and cover are all highly correlated to maximum temperature (> 0.70) Minimum temperature is moderately negative correlated to elevation and precipitation (-0.67). Vegetation height and cover are also highly correlated to slope (> 0.70). These correlations may make interpretation of permutation importance difficult between variables like vegetation height and vegetation cover. With this in mind, permutation importance, represented as a percentage, shows some trends amongst the nine species models. The radar vegetation index proved to be a consistently important variable, showing the applicability of this spaceborne vegetation metric to distinguish suitable bird habitat in Mediterranean ecosystems. (Fig. 3). The largest permutation importance values are for maximum temperature in the two species restricted to Santa Cruz Island, the warmest of the three islands modeled. Vegetation height had high permutation importance for Hutton’s Vireo and Chipping Sparrow, two birds that in previous research have been known to prefer woodlands over scrubland or grasslands^34,35^. The Channel Islands subspecies of the Orange-crowned Warbler often nests on wooded slopes, in gullies, canyons, and along steep banks^36^. Among the nine species modeled, slope had the highest permutation importance in the Orange-crowned Warbler model, reflecting the biological relevance of this variable (Fig. 3).

**Fig. 4:**
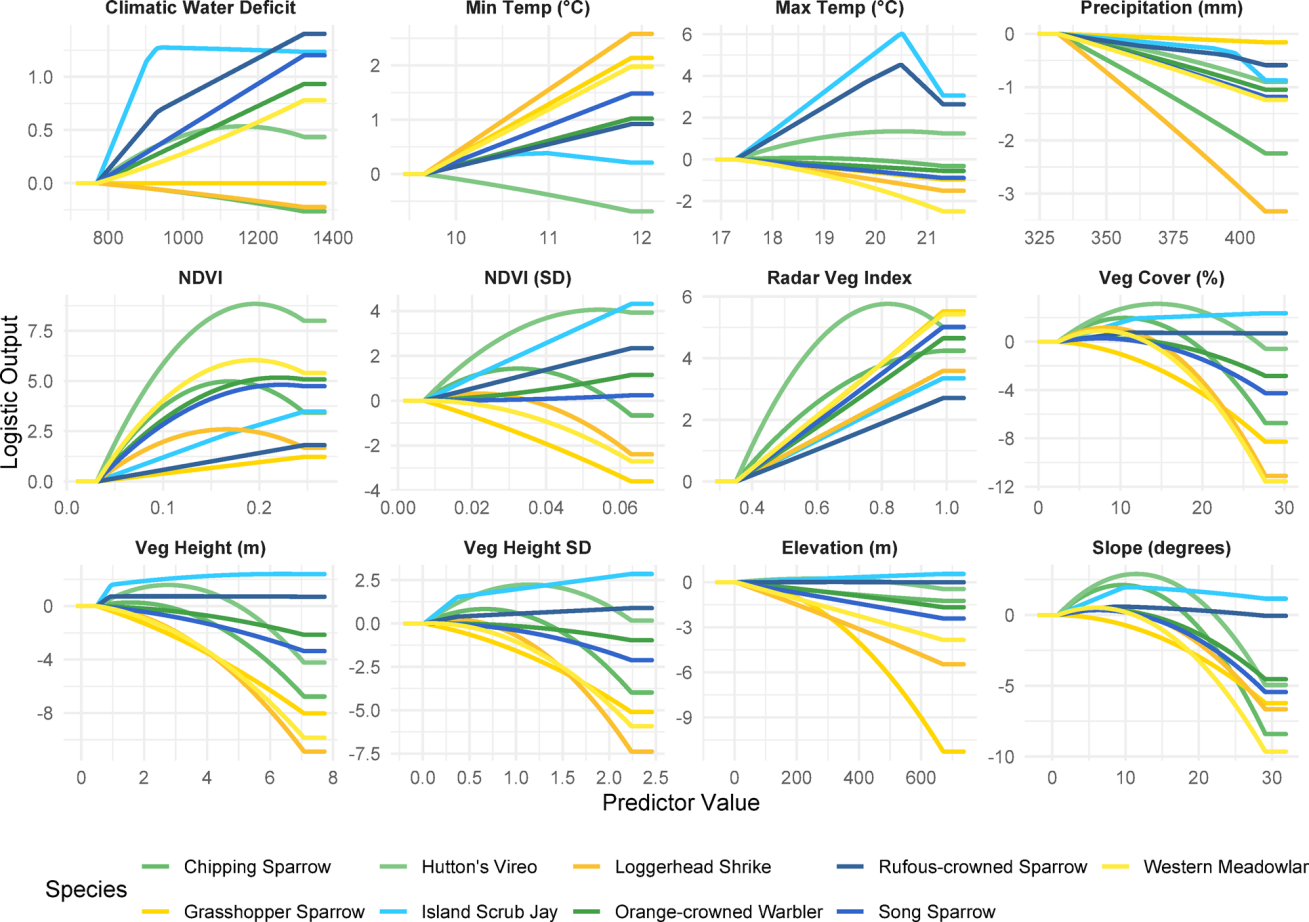
Response curves for each of the nine species representing logistic output of a model built using only one of the individual variables. Species are grouped roughly by habitat preferences with grassland birds in yellow, shrubland birds in blue and woodland birds in green.

Maxent has been shown to be robust against multicollinearity in predictor variables when it comes to measures of performance including AUC^37^. Response curves based on models built using one individual variable can help with interpretation when variables are highly correlated^38^. Overall, patterns in habitat preferences in response curves for our models show fidelity to what are known habitat preferences (i.e. grassland, scrubland and woodland bird species) in CHIS or on the mainland of California. Grassland birds (i.e. Grasshopper Sparrow, Loggerhead Shrike, and Western Meadowlark) show reduced suitability at higher elevations, higher vegetation heights, higher variability of vegetation heights, and higher variability of NDVI values (Fig. 4). These are consistent characteristics with grassland habitats, though it is important to note that not all these variables had high permutation importance for all grassland species. Response curves of grassland bird species models also show increased habitat suitability in areas that experience less variability in temperature from minimum to maximum (Fig. 4). Results are more nuanced between woodland and scrubland birds. However, response curves indicate that scrubland birds prefer drier, hotter environments than other species according to climatic water deficit and maximum temperature (Fig. 3). Specific species results are also consistent with what is found in the literature. For example, suitable breeding habitat for Hutton’s Vireo throughout California is described as arborescent chaparral^34^. Hutton’s Vireo response curve for vegetation height peaks at ~ 3 m (Fig. 4), much taller than the average maximum vegetation height measured during field vegetation surveys throughout the park (~ 1.13 m). Vegetation height also proved to have the highest permutation importance for the Hutton’s Vireo model.

### Future climate projections

The comparison of correlation matrices between present day and future scenarios showed maximum collinearity shifts of ~ 0.3 mostly in relationship to CWD which in present day climate is not highly correlated with any variables. The potential for this collinearity shift in future scenarios could be due to the likely overestimation of the rate of increase in PET under warming conditions calculated using the Priestley-Taylor equation^39^. However, given the moderate shift in collinearity we determined that it would have a small influence on model projections^37^.

Current climate conditions based on the PRISM observations show that Santa Cruz Island experiences higher maximum temperatures than the other two islands and has a strong gradient of annual precipitation with much of it falling in the northwestern portion of the island leaving the eastern and southern portion of the island drier. San Miguel Island is overall cooler and wetter than the other two islands with Santa Rosa having a relatively dry and cool climate with more precipitation falling over its higher elevations. Ensemble means from both the RCP 4.5 and RCP 8.5 scenarios project a generally warmer and wetter future climate for the Channel Islands. Notably, projections indicate that the near-future climate of Santa Rosa Island will become increasingly similar to the current climate of Santa Cruz Island, characterized by rising temperatures and enhanced precipitation, particularly at higher elevations. Both scenarios also suggest that San Miguel Island, historically the coolest of the three, will experience greater increases in both minimum and maximum temperatures compared to Santa Rosa and Santa Cruz. Precipitation is expected to decrease slightly over San Miguel and increase over much of Santa Cruz Island. The four models which stood out in the novelty analysis happen to be the four models which were selected as representative of the range of the full 10 model ensemble in California’s Fourth Climate Change Assessment (Supplemental Note 1;^40^. HadGEM2-CC was labeled as the “warm/dry” model and therefore predicts a near future climate CHIS in RCP4.5 which is analogous to present day climate. CNRM-CM5 which projects the most novel climate for the RCP4.5 scenario is labeled as the “cool/wet” model. CanESM2 projections in the RCP8.5 scenario led to a highly novel environment for all three islands and was labeled as the “average” model by the Fourth Climate Change Assessment^40^. And finally, MIROC5 which still shows some analogous climate in regions of Santa Rosa and Santa Cruz in the RCP 8.5 scenario is the one model deemed most different from the other three (Supplemental Note 1).

The comparison between the full climatic range of the five non-endemic species and the climatic envelope available in the Channel Islands revealed that the Channel Islands occupy the warm, dry end of what is tolerated by these species across their full ranges (Supplemental Material Fig. 2). Island mean annual temperature (median ~ 14.7 °C) falls well above the full-range median for most species (ranging from − 0.4 °C for Chipping Sparrow to 17.5 °C for Hutton’s Vireo), though it remains within the full range of values each species occupies. Similarly, island climatic moisture index values are negative (median ~ -45 mm), reflecting the dry Mediterranean climate of the islands, while full-range CMI extends into strongly positive values for all species. Mean annual precipitation on the islands (104–698 mm, median ~ 403 mm) corresponds to the lower-to-mid portion of each species’ full precipitation range. Taken together, these results suggest that our study area captures a climatically coherent and internally consistent portion of each species’ realized niche. Additionally, most climate models predict a warmer future for the islands with increases in climatic water deficit (equivalent to decreases in climatic moisture index), meaning that the species are likely to experience a future climate on the islands that pushes the boundary of even their full range climatic niche.

### Suitability maps present and future

**Fig. 5:**
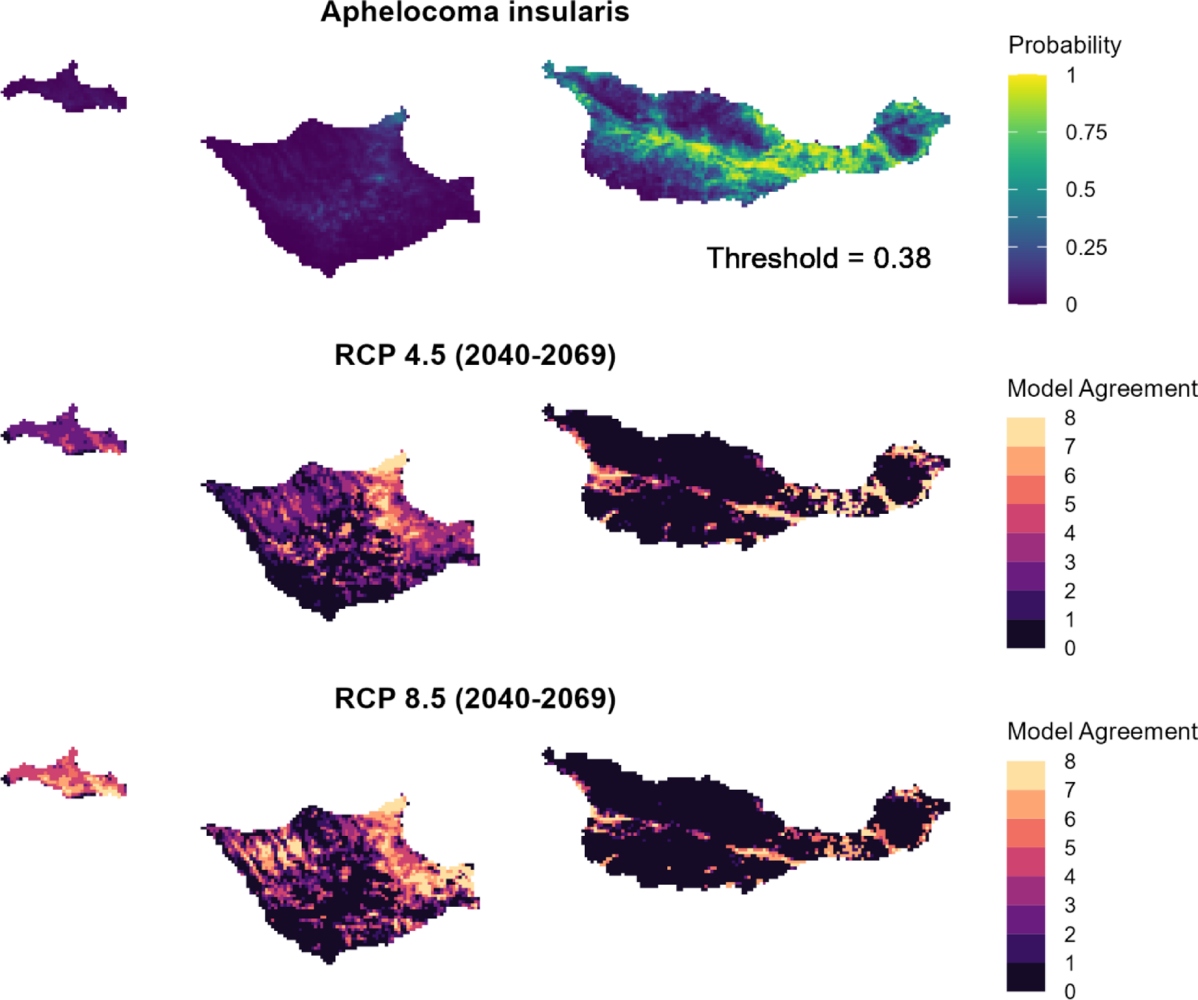
A summary of spatial Maxent model results for Island Scrub Jay (*A. insularis*). The top map represents predicted probability of presence based on present day environmental metrics ranging from 0 (unsuitable) to 1 (highly suitable) in dark blue to yellow. The bottom 2 maps represent cumulative binary maps based on a suitability threshold of 0.38 with black representing areas where all models agree that the area is unsuitable in future scenarios. The following dark to light colors represent how many models agree that the area will be at or above the 0.38 suitability threshold in the two near future scenarios. These maps were generated using the R package ggplot2^42^.

Suitability maps were created for all nine species under current conditions and future climate conditions. We explored specific results for the one single-island endemic species, the Island Scrub Jay to illustrate the interpretation and applications of these maps. Maps of current and future habitat suitability for the other eight species are available in Supplemental Material (Supplemental Material Figs. 3–10). Importantly, translocation is being investigated as a climate adaptation strategy for this species^41^. The present-day scenario shows predicted probability of presence for the northern facing slopes of the Santa Cruz Island and the central valley that runs East to West across the island which aligns with the current range of scrubland and oak woodland habitat (Fig. 5). The 10-percentile presence threshold used to create binary maps for this species is 0.38. Both the RCP 4.5 and RCP 8.5 near-future scenarios show strong model agreement in projecting a reduction in climatically suitable habitat for the Island Scrub Jay on Santa Cruz Island based on the established suitability threshold. Concurrently, as San Miguel and Santa Rosa Islands warm to resemble the current climate of Santa Cruz Island, both scenarios predict an expansion of suitable habitat onto these islands (Fig. 5). This expansion is more pronounced under the RCP 8.5 scenario, which also shows higher model agreement across large areas of San Miguel and Santa Rosa. The distance between Santa Cruz and Santa Rosa islands, ~ 9 km, is too far for unaided dispersal by Island Scrub Jays but a Santa Rosa population could be established through human-assisted dispersal^41^. Further research into potential pressures of competition and predation on endemic Santa Rosa species would be needed to determine the full implications of this assisted dispersal.

Future climate scenarios project a reduction in the extent of suitable habitat for the majority of the nine breeding landbird species examined in this study (Table 2). The most substantial declines are observed for two woodland species, Chipping Sparrow and Hutton’s Vireo, with Maxent models predicting less than 1 km² of suitable habitat remaining across all islands and scenarios, except for Hutton’s Vireo on Santa Cruz Island under RCP 4.5 (1.75 km²). For both species, precipitation and minimum temperature were the most influential climate variables (Fig. 3), and their response curves reveal reduced suitability under more extreme values of precipitation and climatic water deficit (CWD) (Fig. 4). Despite projected increases in total precipitation, CWD is also expected to rise due to shifts in precipitation timing—fewer wet days, more intense storms, and longer dry seasons^40^. These patterns align with broader trends of California vegetation becoming shorter and denser in response to increased water stress^28^, potentially leading to declines in woodland habitats and expansion of scrub shrublands. In contrast, the Grasshopper Sparrow shows slight increases in suitable habitat area across all islands and scenarios. It is important to note that this predicted increase due to climatic changes could be lessened by the continued transition away from grasslands towards native shrublands throughout the islands. Additionally, the two species currently restricted primarily to Santa Cruz Island, Island Scrub Jay and Rufous-crowned sparrow, are projected to expand their ranges to San Miguel and Santa Rosa Islands under future climate conditions. To date, dispersal for both species has likely been limited by scrubland recovery from grazing on other islands. Specifically, Rufous-crowned Sparrow has been found to be susceptible to habitat fragmentation^43^. Additionally, little postbreeding wandering has been observed in both species, limiting their dispersal from breeding sites^44,45^. We focused subsequent analyses on the six species projected to show consistent declines across all three islands (Chipping Sparrow, Hutton’s Vireo, Loggerhead Shrike subspecies, Orange-crowned Warbler subspecies, Song Sparrow, and Western Meadowlark), as these represent the greatest conservation concern for long-term avian biodiversity on the Channel Islands.

**Table 2:** The area in km^2^ of suitable habitat for 3 scenarios (present day and future climate in 2040-2069 for RCP 45 and RCP 85) based on the 10-percentile presence threshold for each species on each island (San Miguel, Santa Rosa, and Santa Cruz).

Species name	San Miguel			Santa Rosa			Santa Cruz		
	Present	RCP45	RCP85	Present	RCP45	RCP85	Present	RCP45	RCP85
Chipping sparrow	0	0	0	132.90	0.14	0.51	72.58	0.80	0
Grasshopper sparrow	19.55	**19.91**	**20.27**	87.31	**93.36**	**98.62**	30.27	**33.11**	**33.92**
Hutton’s vireo	0.36	0	0	61.12	0.23	0.07	81.18	1.75	0.36
Island Scrub Jay	0	0	**5.47**	0.29	**12.91**	**27.79**	91.03	19.47	11.31
Loggerhead shrike	0.51	0	0	145.37	6.93	8.61	52.01	3.94	1.09
Orange-crowned Warbler	18.45	12.03	11.89	121.52	21.37	21.37	79.58	33.62	29.83
Rufous-crowned sparrow	0	**9.41**	**19.69**	1.82	**97.01**	**163.90**	108.68	**138.22**	**138.00**
Song sparrow	19.33	16.34	16.25	126.26	37.35	37.27	77.61	39.46	36.25
Western Meadowlark	19.69	12.11	10.58	140.92	27.13	21.30	42.16	13.64	13.49

Bold values represent increases in suitable habitat area from present to future scenarios.

**Fig. 6:**
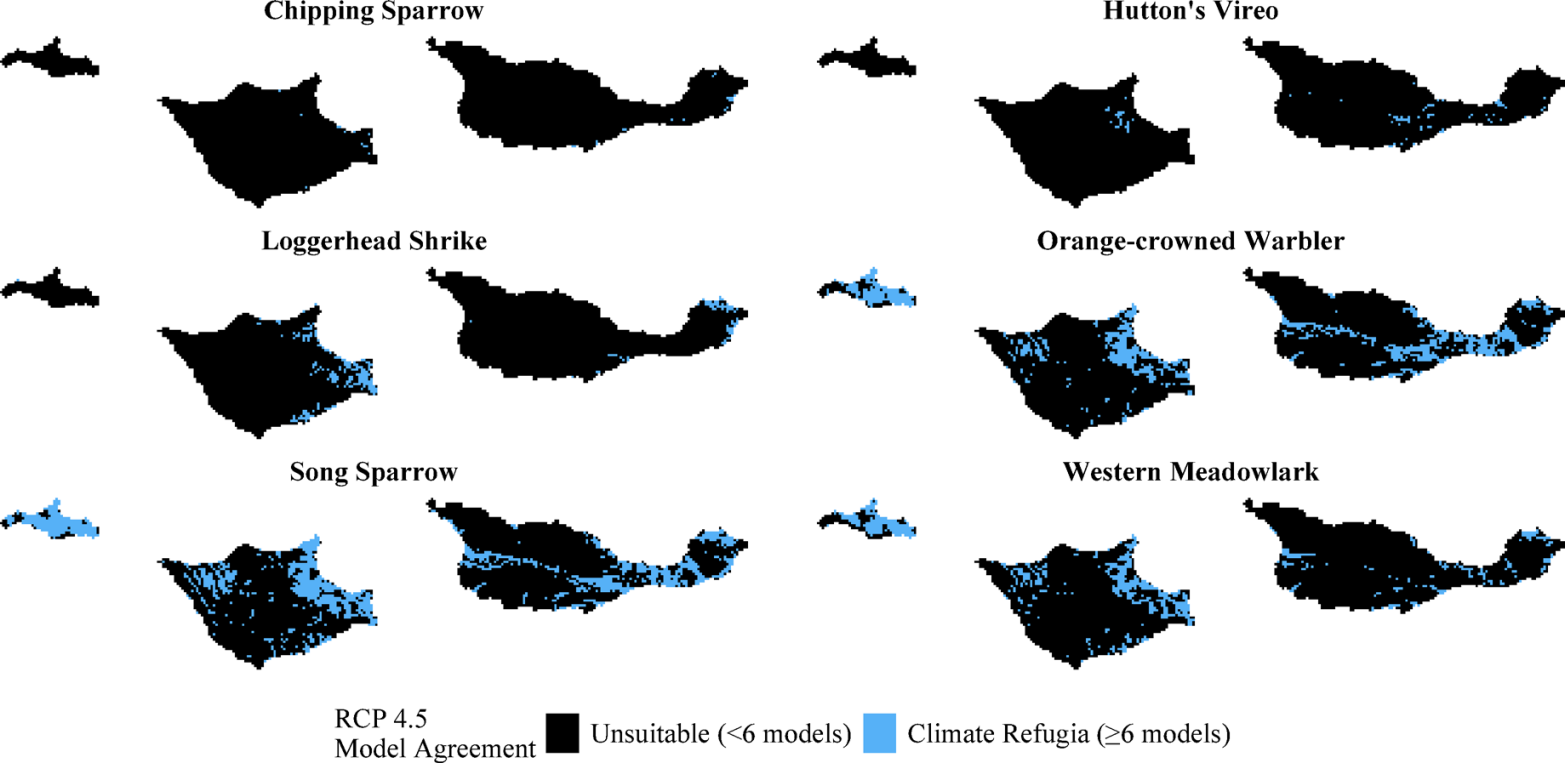
Projected climate-change refugia for six landbird species on the northern Channel Islands, where ≥ 6 of 8 CMIP5 models agree that suitable climatic conditions will persist through 2070 under RCP 4.5. Refugia are shown in blue, representing zones of high model agreement, while areas in black indicate lower agreement (< 6 models). These spatial patterns provide insights into potential strongholds for avian biodiversity under climate change and can inform long-term conservation planning. These maps were generated using the R package ggplot2^42^.

Maps of species-specific climate change refugia, or areas where at least six of the eight RCP 4.5 CMIP5 models agree that suitable climate conditions will persist into 2069, highlight key zones of conservation interest across the northern Channel Islands (Fig. 6). The RCP 4.5 scenario was used to identify climate refugia given the high level of climate novelty in the RCP 8.5 scenario results. Extrapolation outside of conditions experienced in the training data could lead to unreliable model prediction and therefore produce spurious suitability estimates. The northeastern corner of Santa Rosa Island emerges as persistent refugia for five of the six species of concern, reinforcing its ecological significance under future climate conditions. Portions of the central valley and NPS-managed areas of Santa Cruz Island are also projected to retain suitable conditions for some species. Interestingly, large contiguous areas on San Miguel Island are identified as climate refugia for three species, suggesting new opportunities for restoration and reintroduction efforts. These refugial zones offer promising targets for climate-smart conservation strategies focused on persistence and resilience under climate change^3^. However, realizing this conservation potential will require active management of vegetation within refugial areas. Vegetation structure and composition were among the strongest predictors in our present-day models and were held constant in future projections. We identify therefore that habitat suitability in the future is contingent on the persistence of compatible vegetation regimes. Refugial areas where climate conditions are projected to remain suitable represent the highest-priority targets for native vegetation protection, restoration, and monitoring, as these are the landscapes most likely to sustain the habitat conditions amenable to the species modeled in this study.

Uncertainty in our projections arises both from model transfer to future climates and from the climate scenarios themselves, including known biases in CMIP5 models and the challenges of statistically downscaling global products to small, topographically complex islands^46^. Although the eight CMIP5 models used here agree on future increases in CWD and temperature, precipitation projections remain highly variable. Additional sources of uncertainty include non-random survey sampling on Santa Cruz Island, the unknown trajectory of vegetation change under future climate conditions and impacts of potential novel interspecies interactions (Supplemental Note 2). Future work should focus on projecting changes of vegetation cover, type and health under varying climate scenarios in the Channel Islands by leveraging models developed across mainland California with similar vegetation cover types and focusing on areas of the Channel Islands that have evaded grazing pressure despite widespread ungulate populations. More systematic bird survey designs across Santa Cruz Island, or approaches that explicitly model detection probability across survey types, would also strengthen future inference and reduce uncertainty arising from non-random sampling.

## Conclusions

Our study demonstrates the utility of integrating GEDI-derived vegetation structure metrics with Landsat reflectance and topographic variables to produce spatially explicit models of vegetation cover and height across the California Channel Islands. The strong performance of our random forest models, particularly their biophysical interpretability and sensitivity to known environmental gradients such as elevation and SWIR1 reflectance, underscores the effectiveness of remote sensing in monitoring vegetation change over time. Importantly, the application of a GAM-based correction using recent ground validation data reduced model bias and enhanced the reliability of our height predictions, though some underestimation of taller vegetation remains. Our results indicate continued ecological recovery on the islands, consistent with long-term trends in NDVI and shifts from non-native grasslands to native woody vegetation following the removal of invasive grazers. Our Maxent results further reveal that habitat specialization and systematic sampling are key to model performance. Models based on expert-derived NPS survey data showed higher AUC scores than those supplemented with spatially-biased citizen science records, however the addition of GBIF data consistently improved Boyce Index performance, highlighting the complementary value of citizen science observations for model calibration. Response curves and spatial trends aligned with known habitat preferences for island-endemic and specialized bird species, reinforcing the relevance of remotely sensed vegetation metrics, particularly RVI for predicting suitable breeding habitat in Mediterranean ecosystems. When projected to future climate scenarios, models highlight both the promise and limits of SDMs under novel conditions. While clamping offers a conservative strategy to reduce overextrapolation, it cannot capture potential non-linear tipping points in species responses to extreme environmental change. The degree of climate novelty, especially in RCP 8.5 scenarios, suggests that caution is warranted in interpreting species responses under high-emission futures. Nevertheless, our findings provide a critical foundation for tracking habitat change and informing conservation planning in the face of climate uncertainty. Continued remote sensing, ground validation, and systematic biodiversity monitoring will be essential to support adaptive management of these ecologically sensitive island systems. Most species experienced a decline in area of suitable habitat on all three islands in both near future scenarios with the exception of the Grasshopper sparrow, Island scrub jay and Rufous-crowned sparrow.

## Methods

### Breeding landbird species data

We compiled annual point count data collected by NPS and partners from 2014 onward for nine priority land bird species. Surveys followed Coonan et al.^47^ protocols and were conducted at established transects sites (survey stations) across the Channel Islands (Fig. 1). On Santa Cruz Island, where land is divided between the Channel Islands National Park and the Nature Conservancy (TNC), 100 additional non-random survey stations established by R. C. Klinger along trails and roads in 2013 and have since been surveyed annually. These years were selected to capture the expanded Santa Cruz survey network and potential effects of herbivore removal. Throughout this study, data from survey stations collected by NPS and partners are referred to as “NPS data”. We collected citizen science data from the Global Biodiversity Information Facility (GBIF) on all nine priority land bird species for the same range of years. Observations were filtered to the breeding season (February through June) to match the seasonal survey effort of the NPS. We additionally set the maximum coordinate uncertainty to 100 m.

### Vegetation structure and cover data

We selected relative height 95 from GEDI’s level 2A^48^ Geolocated Elevation and Height Metrics and cover from GEDI’s level 2B^48^ Canopy Cover and Vertical Profile Metrics products to describe vegetation height and percent cover over CHIS in Google Earth Engine (GEE)^49^. To accurately train the vegetation height and cover models, we filtered and processed GEDI waveforms collected between November and March of the years 2019 to 2022 based on quality and sensitivity properties (Supplemental Methods). These waveforms served as training data in a 250-tree random forest model with the square root of the number of total variables used per split. The variables used in the random forest were all surface reflectance values from Landsat 8 Level 2, Collection 2, Tier 1 imagery along with the 30 m NASA DEM layer.

The random forest models used to predict vegetation cover and vegetation height were evaluated internally using an out of bag error estimate. We determined variable importance as the sum of decrease in Gini impurity index over all trees in the individual models. Independent field-based vegetation data collected by Channel Islands National Park (CHIS) staff from 2021 to 2024 were used solely for external validation of the vegetation height predictions. Vegetation surveys were conducted on all five islands using a line-point intercept method, with 30 m transects and species identity and vegetation height recorded every 30 cm along each transect^50^. Predicted vegetation height from the random forest models was compared against these field measurements to assess model performance across islands. Given that the relationship between modeled and actual height on the ground will likely be non-linear, we corrected the vegetation height layer using maximum height data along the transects in a generalized additive model (GAM). RMSE were calculated before and after the GAM correction using NPS field data and compared to the RMSE of a previously produced 10 m global vegetation height model^25^. The NPS data only covers the eastern portion of Santa Cruz Island, importantly leaving out areas with some of the tallest vegetation. To test if the GAM correction underestimates height in this area, we collected independent vegetation height data in Santa Cruz Island Conservancy at 47 random sites placed within 200 m of accessible roads stratified by ESA Land Cover types (Shrubland, Tree Cover, Grassland) using a graduated survey rod for shrubs and grasses and a range finder for taller vegetation. We used these points to calculate a RMSE and Bias on the corrected vegetation height model for this additional data.

The NDVI composite used in the species habitat suitability models was created from a median of cloud masked Landsat images from 2013 to 2023 when looking at months November through March. To represent the variation of NDVI values across the landscape we used a 3 × 3 pixel kernel to calculate the standard deviation of Landsat derived NDVI values in GEE. This kernel size was selected to fully capture the surrounding area of an individual pixel. Radar Vegetation Index (RVI), another predictor variable in the habitat suitability models, quantifies vegetation structure and biomass by measuring the randomness of microwave scattering. RVI ranges from 0 to 1, with low values indicating smooth bare surfaces and higher values reflecting increasing vegetation growth, biomass, and water content over the growing season^51^. To calculate RVI we fetched dual-polarization C-band (5.405 GHz) Synthetic Aperture Radar (SAR) from the Sentinel-1 mission in GEE (Supplemental Methods). Vertical-vertical (VV) polarization, used in RVI calculation (Supplemental Methods), represents backscatter where the radar transmits a vertically polarized wave and receives the vertically polarized signal which captures reflections of vertical structure. Vertical-horizontal (VH) polarization, also used in RVI calculation, is where the radar transmits a vertically polarized wave but receives a horizontally polarized signal, which makes it useful for detecting features like vegetation as it highlights complex scattering caused by branches and leaves^52^. We also collected the European Space Agency’s 2021 global land cover map derived from Sentinel-1 and Sentinel-2 data which has the following cover types over CHIS: tree cover, shrubland, grassland, cropland, built-up and bare/sparse vegetation^53^.

We selected topographic and vegetation metrics following a two-step process: we first screened candidate variables for biological relevance to the target species with input from experts (Supplemental Methods), then assessed collinearity using variance inflation factors (VIF), iteratively removing variables to reduce VIF where possible while retaining ecologically meaningful predictors. The only three variables retained despite elevated VIF were vegetation height, standard deviation of vegetation height and percent vegetation cover. We chose to retain these because model performance declined for several species when either was removed, and because together they capture complementary aspects of vegetation structure, mean canopy height and heterogeneity, and percent of ground covered by vegetation in a pixel, that are not redundant in a biological sense. This is especially important given that characterizing vegetation composition across the islands is a primary objective of this study.

### Climate data

The Basin Characterization Model (BCMv8), developed by USGS scientists in cooperation with California Department of Water Resources, is a water balance model used to assess fine scale hydraulic response to changes in climate in California^54^. BCM data was chosen over other North American dynamical downscaling climate products (i.e. ClimateNA) after qualitative assessment of spatial trends in minimum and maximum temperature on the islands using Landsat thermal band observations. To model suitable climatic ranges for the landbirds species we chose three of the four climate inputs to the BCM; monthly minimum and maximum temperature (averaged over water years) and precipitation (totaled over water year), along with one hydrologic output from the BCM, climatic water deficit (CWD)^55^. CWD represents the annual evaporative demand that exceeds available water, or PET – actual evapotranspiration, summed annually. The present-day habitat suitability models were built using observed climate and modelled hydrologic normals for the 1990–2019 period. Due to their small size and restricted climate observations, there are no data from BCM v8 available for Santa Barbara Island and Anacapa Island, therefore the study focuses solely on Santa Cruz Island, Santa Rosa Island and San Miguel (Fig. 1).

BCM v8 was applied to downscaled projections of precipitation, minimum temperature, and maximum temperature from 10 CMIP5 models under two warming scenarios (RCP 45 and RCP 85). For this study, only models which had full datasets for both scenarios in the near-term future (2040–2069) were implemented leaving the following 8 models: ACCESS1.0, CanESM2, CESM1-BGC, CMCC-CMS, GFDL-CM3, HadGEM2-CC, HadGEM2-ES, and MIROC5. The 10 original models were chosen based on GCM historical performance in representing mean seasonal spatial patterns in the Southwestern US, amplitude of seasonal cycle, diurnal temperature range, annual to decadal scale variance and regional teleconnections to El Nino Southern Oscillation^40^. To capture a more robust range of possible climate futures, each species model was projected to all 16 climate model outputs. Additionally, we implemented the delta change method of bias correction on climate normals produced from the 8 models for the 1980–2009 period compared to observed climate normals from the same period^37,56^ discovered that Maxent was robust to the inclusion of collinear variables. However, projection away from the calibration zone in either time or space is impacted by novelty and collinearity shifts. Because removing highly correlated variables during calibration does not eliminate the risk of such shifts, changes in correlation structure were assessed prior to modeling. To assess collinearity shift, we calculated the mean absolute difference between a correlation matrix of present day climate variables and one of future climate variables for the RCP 45 and RCP 85 scenarios^37^. Additionally, we calculated a standardized novelty score based on Mahalanobis distance, for each of the 16 climate models outputs to determine areas where future climate conditions will be non-analogous to the present Channel Islands climate^57^. This extrapolation detection method considers both deviations from the mean and the correlation between variables and will determine the uncertainty in the Maxent model projections into the near future.

Five of the nine study species are non-endemic, with broad continental distributions that extend well beyond the Channel Islands. Models built from such a geographically restricted region risk capturing only a truncated portion of each species’ full climatic tolerance, potentially compromising the reliability of future projections if island populations can in fact persist under climate conditions not represented in our training data. To evaluate this concern, we compared the climatic space occupied by our study area against full-range species distribution models developed by the National Audubon Society^58^, which were built from occurrence data spanning each species’ entire North American range. More specifically, we extracted the three bioclimatic variables most analogous to those used in our models (Climatic Moisture Index, Mean Annual Temperature, and Mean Annual Precipitation) from the full-range models for each species and compared their distributions to climate conditions available across the Channel Islands.

### Maxent modelling

To optimize model parameters for each species, we used the ENMevaluate tool in the *ENMeval* R package to calibrate Maxent models based on National Park Service (NPS) data alone and combined NPS + GBIF occurrence records^59^. Model tuning involved a 10-fold cross-validation procedure to evaluate combinations of feature classes and regularization multipliers. When the optimal parameters differed between the NPS and NPS+GBIF models, the model with fewer coefficients was selected to reduce overfitting and enhance transferability to future climate scenarios^60^. Final models were then trained using the spatialMaxent package^61^, which employs 10-fold spatial cross-validation, partitioning occurrence data into geographically distinct blocks to account for spatial autocorrelation and prevent overly optimistic performance estimates. Habitat suitability maps were generated by averaging predicted probabilities of presence across the 10 submodels. Model performance was assessed using the average area under the receiver operating characteristic curve (AUC) and Boyce Index, along with the standard deviation among submodels to estimate uncertainty. Holding all the topographic and vegetation metrics the same (Supplemental Methods), the trained model results are then applied to near future (2040–2069) climate projections for the RCP 4.5 and RCP 8.5 scenarios for 8 different CMIP5 models. We then produced binary maps of habitat suitability for present and future climate conditions using the 10th percentile training presence threshold, which corresponds to the predicted suitability value above which 90% of training points occur^62^. To evaluate model agreement in suitable habitat under future climate conditions and identify potential climate change refugia areas, cumulative binary maps were produced for RCP 4.5 and RCP 8.5. Changes in suitable habitat were assessed by comparing the area (in km²) of suitable habitat in the present day to areas where at least 6 CMIP 5 models agree there will be suitable habitat under future projections for each of the three main islands. Climate change refugia are identified as the areas in which at least 6 CMIP 5 models agree there will suitable habitat under the future RCP 4.5 scenario. These areas represent where a majority (two-thirds) of climate models agree on future suitability for the species. Given the variability present in the 8 CMIP models used, calculating areas where all models agree would be overly pessimistic in terms of likely climate refugia persistence.

## Supplementary Information

Below is the link to the electronic supplementary material.

Supplementary Material 1

## Data Availability

Climate data can be downloaded here: https://www.sciencebase.gov/catalog/item/61e069f3d34e8911d9fe9dba. All remote sensing data is available in Google Earth Engine Repositories cited in the article. Vegetation data collected for this study will be made available in a Dryad repository and data collected by the National Park Service (vegetation surveys and landbird point counts) can be requested through the research permit and reporting system: https://irma.nps.gov/RPRS/. All code used in this study will be made available in a GitHub repository upon publication.
