# Supplementary material for: Sensor fusion and downscaled climate projections reveal climate refugia in the California Channel Islands
Source: Sci Rep. 2026 Jun 8;16:25984. doi: 10.1038/s41598-026-56345-4 (PMC13487183; doi:10.1038/s41598-026-56345-4)
Supplement: Supplementary file 1 [file 41598_2026_56345_MOESM1_ESM.docx]

# **Sensor fusion and downscaled climate projections reveal climate refugia in the California Channel Islands**

Erica M. Gallerani^1^, Stacey Ostermann^2^, Cameron B. Williams^2^, G. Andrew Fricker^3^, Kyle C. Cavanaugh^1^, and Thomas W. Gillespie^1^

^1^ Department of Geography, University of California Los Angeles, Los Angeles, California, USA

^2^ Mediterranean Coast Network, NPS Inventory & Monitoring Program, 401 West Hillcrest Dr., Thousand Oaks, CA 91360, USA

^3^ Division of Natural Resources, Channel Islands National Park, Ventura, CA 93001

^4^ Social Sciences Department, California Polytechnic University, San Luis Obispo, California USA

**Supplemental Material**

| Table 1. List of 9 study species with reasons for prioritization. | | |  |
| --- | --- | --- | --- |
| Species Name | Common Name | Reason for Prioritization | |
| *Spizella passerina* | Chipping Sparrow | Declining | |
| *Ammodramus savannarum* | Grasshopper Sparrow | Low density, grassland species | |
| *Vireo huttoni* | Hutton’s Vireo | Declining | |
| *Aphelocoma insularis* | Island Scrub Jay | Threatened, endemic | |
| *Lanius ludovicianus anthonyi* | Loggerhead Shrike | Declining, island subspecies, endemic | |
| *Leiothlypis celata sordida* | Orange-crowned Warbler | Island subspecies, endemic | |
| *Aimphila ruficeps obscura* | Rufous-crowned Sparrow | Restricted to one island, endemic | |
| *Melospiza melodia* | Song Sparrow | Declining | |
| *Sturnella neglecta* | Western Meadowlark | Grassland species, declining | |
|  | | |  |

Figure 1. The GAM corrected relative height 95 model derived from spaceborne lidar, Landsat imagery, and elevation data for all five islands. This map was generated using ArcGIS Pro Software Version 3.4.0 (ESRI 2025).

Figure 2. Violin plots showing the comparison between full-range distribution models for the five non-endemics species and the climatic niche available on the Channel Islands. The blue plots represent the full-range of climate tolerance for each species while the orange plots represent the Channel Islands values. (CHSP = Chipping Sparrow, GRSP = Grasshopper Sparrow, HUVI = Hutton’s Vireo, SOSP = Song Sparrow, WEME = Western Meadowlark).

Figure 3: A summary of spatial Maxent model results for Rufous-crowned sparrow (*A. ruficeps*). The top map represents predicted probability of presence based on present day environmental metrics ranging from 0 (unsuitable) to 1 (highly suitable) in dark blue to yellow. The bottom 2 maps represent cumulative binary maps based on a suitability threshold of 0.39 with black representing areas where all models agree that the area is unsuitable in future scenarios. The following dark to light colors represent how many models agree that the area will be at or above the 0.39 suitability threshold in the two near future scenarios. These maps were generated using the R package ggplot2 (Wickham 2016).

Figure 4: A summary of spatial Maxent model results for Grasshopper Sparrow (*A. savannarum*). The top map represents predicted probability of presence based on present day environmental metrics ranging from 0 (unsuitable) to 1 (highly suitable) in dark blue to yellow. The bottom 2 maps represent cumulative binary maps based on a suitability threshold of 0.48 with black representing areas where all models agree that the area is unsuitable in future scenarios. The following dark to light colors represent how many models agree that the area will be at or above the 0.48 suitability threshold in the two near future scenarios. These maps were generated using the R package ggplot2 (Wickham 2016).

Figure 5: A summary of spatial Maxent model results for Loggerhead shrike (*L. ludovicianus*). The top map represents predicted probability of presence based on present day environmental metrics ranging from 0 (unsuitable) to 1 (highly suitable) in dark blue to yellow. The bottom 2 maps represent cumulative binary maps based on a suitability threshold of 0.27 with black representing areas where all models agree that the area is unsuitable in future scenarios. The following dark to light colors represent how many models agree that the area will be at or above the 0.27 suitability threshold in the two near future scenarios. These maps were generated using the R package ggplot2 (Wickham 2016).

Figure 6: A summary of spatial Maxent model results for Orange-crowned warbler (*L. celata*). The top map represents predicted probability of presence based on present day environmental metrics ranging from 0 (unsuitable) to 1 (highly suitable) in dark blue to yellow. The bottom 2 maps represent cumulative binary maps based on a suitability threshold of 0.36 with black representing areas where all models agree that the area is unsuitable in future scenarios. The following dark to light colors represent how many models agree that the area will be at or above the 0.36 suitability threshold in the two near future scenarios. These maps were generated using the R package ggplot2 (Wickham 2016).

Figure 7: A summary of spatial Maxent model results for Song Sparrow (*M. melodia*). The top map represents predicted probability of presence based on present day environmental metrics ranging from 0 (unsuitable) to 1 (highly suitable) in dark blue to yellow. The bottom 2 maps represent cumulative binary maps based on a suitability threshold of 0.35 with black representing areas where all models agree that the area is unsuitable in future scenarios. The following dark to light colors represent how many models agree that the area will be at or above the 0.35 suitability threshold in the two near future scenarios. These maps were generated using the R package ggplot2 (Wickham 2016).

Figure 8: A summary of spatial Maxent model results for Chipping Sparrow (*S. passerina*). The top map represents predicted probability of presence based on present day environmental metrics ranging from 0 (unsuitable) to 1 (highly suitable) in dark blue to yellow. The bottom 2 maps represent cumulative binary maps based on a suitability threshold of 0.33 with black representing areas where all models agree that the area is unsuitable in future scenarios. The following dark to light colors represent how many models agree that the area will be at or above the 0.33 suitability threshold in the two near future scenarios. These maps were generated using the R package ggplot2 (Wickham 2016).

Figure 9: A summary of spatial Maxent model results for the Western Meadowlark (*S. neglecta*). The top map represents predicted probability of presence based on present day environmental metrics ranging from 0 (unsuitable) to 1 (highly suitable) in dark blue to yellow. The bottom 2 maps represent cumulative binary maps based on a suitability threshold of 0.31 with black representing areas where all models agree that the area is unsuitable in future scenarios. The following dark to light colors represent how many models agree that the area will be at or above the 0.31 suitability threshold in the two near future scenarios. These maps were generated using the R package ggplot2 (Wickham 2016).

Figure 10: A summary of spatial Maxent model results for Hutton’s Vireo (*V. huttoni*). The top map represents predicted probability of presence based on present day environmental metrics ranging from 0 (unsuitable) to 1 (highly suitable) in dark blue to yellow. The bottom 2 maps represent cumulative binary maps based on a suitability threshold of 0.21 with black representing areas where all models agree that the area is unsuitable in future scenarios. The following dark to light colors represent how many models agree that the area will be at or above the 0.21 suitability threshold in the two near future scenarios. These maps were generated using the R package ggplot2 (Wickham 2016).

**References**

Esri Inc. *ArcGIS Pro* (Version 3.4) [Software]. <https://www.esri.com/en-us/arcgis/products/arcgis-pro/overview> (2025).

Wickham H. *ggplot2: Elegant Graphics for Data Analysis*. Springer-Verlag New York. ISBN 978-3-319-24277-4. [https://ggplot2.tidyverse.org](https://ggplot2.tidyverse.org/). (2016).

**Supplemental Note 1**

Several steps were taken to prevent overfitting in our species models to ensure transferability to the future climate scenarios. More complex models are likely to do a better job of explaining training data but may be overfit and do a poor job at projecting models to future climate scenarios (Araujo et al. 2019). However, projecting models to environments that lay outside of the values used to calibrate the model can be problematic as it is difficult to predict how species will respond to these novel environmental conditions. The novelty analysis in this study revealed that for RCP45, CNRM-CM5 predicts the most novel climate for the Channel Islands while HadGEM2-CC projections led to a near future climate which is analogous to the present for most of the islands. CanESM2 projections in the RCP85 scenario led to a highly novel environment for all three islands while MIROC5 predicts some analogous climate in regions of Santa Rosa and Santa Cruz with moderate novelty scores for the remaining islands. The “average” model being highly novel in the RCP 85 scenario shows that projections into this more severe climate change future may be less reliable. This is especially true for models which predict a much wetter future for CHIS. For the RCP 45 scenario, standardized novelty scores suggest that results on Santa Rosa Island will be the most reliable. The way that Maxent addresses the issue of projection is clamping, wherein instead of simply extrapolating response curves into novel predictor variable values it treats those novel values the same as the extremes of the calibration zone (Merow et al. 2013). This is a more conservative assumption and likely more realistic than extrapolation, however it relies on an untested assumption that the novel environments do not contain tipping points for species response. For example, clamping maximum temperature and precipitation response is likely reasonable for CHIS species as most response curves experience negative trends at the extremes of the calibration values. However, for CWD and minimum temperature it is possible that the slope of the species response curves could change (from increasing to decreasing) as values exceed those seen in present day climate, something not captured by clamping. Maximum temperature and precipitation are the two variables that contribute the most to standardized novelty scores in most if not all future model scenarios. Given this fact, while there remains some uncertainty in future projections, it is likely that clamping did not have a large effect on most model outcomes.

**References**

Araújo, M. B., Anderson, R. P., Barbosa, A. M., Beale, C. M., Dormann, C. F., Early, R., Garcia, R. A., Guisan, A., Maiorano, L., Naimi, B., O’Hara, R. B., Zimmermann, N. E. & Rahbek, C. Standards for distribution models in biodiversity assessments. *Science Advances.* **5** (2019).

Merow, C., Smith, M. J., & Silander, J. A. A practical guide to MaxEnt for modeling species’ distributions: What it does, and why inputs and settings matter. *Ecography*, **36**, 10, 1058–1069. <https://doi.org/10.1111/j.1600-0587.2013.07872.x> (2013).

**Supplemental Note 2**

Besides the uncertainty associated with projecting model results to novel environments, there is also uncertainty inherent in the climate projections themselves. Sources of this uncertainty include systematic biases in the CMIP 5 models and assumptions of methods used to downscale those models to biologically relevant scales over small islands (Gallerani et al. 2025). The 8 models used in this study consistently show increases in CWD, minimum temperature and maximum temperature for the Channel Islands to varying degrees. However, the models have higher variation in their predictions of precipitation over CHIS. Recent studies have shown that newer CMIP6 models simulate more realistic climate feedbacks in the Pacific (Cai et al. 2021, Planton et al. 2021). Therefore, projecting these models to future scenarios produced by CMIP 6 models may reduce the uncertainty in future precipitation over the California Channel Islands. Additionally, there are significant drawbacks from using statistical downscaling over small topographically complex islands (Gallerani et al. 2025). Exploring the results using different methods of downscaling global climate models could allow for a more robust look at potential future climate scenarios in CHIS.

Additional uncertainty in our habitat suitability models may stem from the non-random sampling structure implemented on the western portion of Santa Cruz Island from 2013 onward. The 100 points placed along roads and trails and in this region could introduce spatial bias, potentially leading to overfitting of model predictions to the central valley of Santa Cruz Island, particularly for species with restricted distributions that are not found on other islands. Future systematic and spatially balanced surveys across Santa Cruz Island, especially targeting the Island Scrub Jay and Rufous-crowned Sparrow, would help validate current model outputs, particularly the prediction of limited habitat suitability in the island’s northern ridges.

A key question not addressed by this study is how vegetation will respond to these changes in future climate conditions. It is difficult to attribute past changes in vegetation in CHIS to changes in climate given the strong history of invasive species removal throughout the park. Without the strong link in the past, it is difficult to extrapolate to the future. However, there is the potential for exploring changes in NDVI in areas that have had consistent native vegetation cover on Santa Cruz Island from the 1980s forward. Additionally, studies of similar areas in mainland California could be extrapolated to predict future changes in parts of the park. One past study looking at *Castilleja mollis* on Santa Rosa Island post herbivore removal found that climate had a significant impact on species recovery (McEachern et al. 2009). To be even more confident on the future model projections, studies should be focused on likely changes to vegetation type, height, density and health (NDVI) in the Channel Islands. Additionally, further research is necessary into the impacts of potentially novel interspecies interactions (i.e. predation and competition) for species who are predicted to have range expansions in the future.

**References**

Cai, W., Santoso, A., Collins, M., Dewitte, B., Karamperidou, C., Kug, J.-S., Lengaigne, M., McPhaden, M. J., Stuecker, M. F., Taschetto, A. S., Timmermann, A., Wu, L., Yeh, S.-W., Wang, G., Ng, B., Jia, F., Yang, Y., Ying, J., Zheng, X.-T., Bayr, T., Brown, J. R., Capotondi, A., Cobb, K. M., Gan, B.¸ Geng, T., Ham, Y.-G., Jin, F.-F.¸ Jo, H.-S., Li, X., Lin, X., McGregor, S., Park, J.-H., Stein, K., Yang, K., Zhang, L. & Zhong, W. Changing El Niño–Southern Oscillation in a warming climate. *Nature Reviews Earth & Environment.* **2**, 628–644 (2021).

Gallerani, E. M., Williams, A. P., Cavanaugh, K. C. & Gillespie, T. W. Uncertainties in Modelling Hawaii’s Future Precipitation and What It Means for Endangered Forest Birds: A Review. *Journal of Biogeography*. **52,** e15121 (2025).

Mceachern, K. A., Thomson, D. M. & Chess, K. A. Climate alters response of an endemic island plant to removal of invasive herbivores. *Ecological Applications* **19**, 1574–1584 (2009).

Planton, Y. Y., Guilyardi, E., Wittenberg, A. T., Lee, J., Gleckler, P. J., Bayr, T., McGregor, S., McPhaden, M. J., Power, S., Roehrig, R., Vialard, J. & Voldoire, A. Evaluating climate models with the CLIVAR 2020 ENSO metrics package. *Bull. Amer. Meteor. Soc.* **102**, 193–217 (2021).

**Supplemental Methods**

We filtered the 25 m waveforms to include those which were collected between November and March of the years 2019 through 2022 and quality filtered by the degrade flag, sensitivity, and quality flag properties. To avoid the inclusion of biased height measurements as training data for the random forest model, we filtered GEDI L2A points that fell outside of 2 standard deviations from the mean vegetation height measured over the Channel Islands. Additionally, we filtered out GEDI L2A points from southern facing slopes using an aspect layer derived from NASA’s merged global digital elevation model (DEM) 1 arc second dataset in GEE (NASA JPL 2020). This filter masked out waveforms collected over aspect values between 150 and 250 as many GEDI L2A waveforms overestimate vegetation height on the steep south/southwestern cliffs of Santa Rosa, San Miguel, Anacapa and Santa Barbara Islands. This left us with over 17,000 training points for the vegetation height random forest model and around 25,000 training points for the percent cover random forest model.

We implemented border noise correction, speckle filtering, and terrain flattening correction as laid out in Mullissa et al. (2021) to the VVVH polarization of both the ascending and descending orbit of the SAR data collected in the interferometric wide (IW) imaging mode. This corrected data was then used to create mean composites of the VV and VH polarizations between November 2022 and March 2023 which were used to calculate the RVI according to the following equation:

$\begin{aligned} RVI=\frac{4\sigma_{VH}^{0}}{\sigma_{VV}^{0}+ \sigma_{VH}^{0}}\#\left( 1 \right) \end{aligned}$

Where $\sigma_{VV}^{0}$ and $\sigma_{VH}^{0}$ represent the polarized backscattering coefficients of the VV and VH radar (Nasirzadehdizaji et al. 2019).

*ALS Topographic Metrics*

We utilized the U.S. Geological Survey DEM for the CHIS to calculate topographic metrics including slope, heat load index and terrain ruggedness. The DEM tiles, derived from lidar point clouds collected in October and November 2010, were processed into a 1/9 arc-second resolution in 2011. In the terra package in R, we mosaiced the DEM tiles into a single layer. We then computed slope using the *terra* package in R, and head load index, representing incident radiation, and terrain ruggedness index (TRI) using the *spatialEco* package in R (Evans et al. 2023). A comprehensive list of topographic and vegetation metrics is provided in Table 2. The variable inflation factors for each metric included in the models are displayed in Table 3.

**Table 2.** List of vegetation and topographic metrics included in the avian habitat suitability models along with the source of the remote sensing data used to derive said metrics.

| **Metric Name** | **Source** | **Reason for inclusion** | |
| --- | --- | --- | --- |
| Normalized difference vegetation index (NDVI) | Landsat | | Proxy for vegetation greenness and productivity; reflects habitat quality and food resource availability for passerines |
| Standard deviation of NDVI (NDVI_SD) | Landsat | | Captures spatial heterogeneity in vegetation productivity; heterogeneous landscapes support greater habitat diversity and species richness |
| Radar vegetation index (RVI) | Sentinel-1 | | SAR-based measure of vegetation structure and biomass; complements optical NDVI by penetrating canopy and remaining unaffected by atmospheric conditions |
| Landcover type (landcover) | Sentinel-1 | | Broad habitat classification capturing dominant vegetation communities; fundamental determinant of species presence and habitat association |
| Percent vegetation cover (cover) | GEDI + Landsat | | Measures canopy closure and understory density; influences nesting habitat suitability, predation risk, and microclimate |
| Relative height 95 (veg_height) | GEDI + Landsat | | Characterizes mean canopy height as a proxy for vegetation structural complexity and successional stage; associated with foraging guild and nesting preferences |
| Standard deviation of relative height 95 (height_sd) | GEDI + Landsat | | Captures vertical structural heterogeneity; areas with variable canopy height support a broader range of foraging and nesting strategies |
| Elevation | ALS | | Important in the characterizations of microclimates and often acts as the primary driver of species distributional limits |
| Slope | ALS | | Influences drainage, soil moisture, and vegetation composition |
| Heat load index (heatload) | ALS | | Integrates aspect and slope to estimate solar radiation and thermal exposure; captures fine-scale variation in thermal environment relevant to thermoregulation and habitat use |
| Terrain ruggedness index (TRI) | ALS | | Quantifies topographic complexity; rugged terrain can provide refugia from disturbance and predation and is associated with habitat heterogeneity |

**Table 3.** List of all metrics included in the avian habitat suitability models and their variable inflation factors (VIF)

| **Metric Name** | **VIF** |  |
| --- | --- | --- |
| Normalized difference vegetation index (NDVI) | 2.47 | |
| Standard deviation of NDVI (NDVI_SD) | 2.94 | |
| Radar vegetation index (RVI) | 2.60 | |
| Landcover type (landcover) | 1.48 | |
| Percent vegetation cover (cover) | 12.65 | |
| Relative height 95 (veg_height) | 13.80 | |
| Standard deviation of relative height 95 (height_sd) | 16.65 | |
| Elevation | 3.14 | |
| Slope | 5.10 | |
| Heat load index (heatload) | 1.33 | |
| Terrain ruggedness index (TRI) | 1.16 | |

References

Evans J.S., & Murphy, M.A. *spatialEco*. R package version 2.0-3, <https://github.com/jeffreyevans/spatialEco> (2023).

Mullissa A., Vollrath A., Odongo-Braun C., Slagter B., Balling J., Gou Y., Gorelick N., & Reiche J. Sentinel-1 SAR Backscatter Analysis Ready Data Preparation in Google Earth Engine. *Remote Sensing*.**13**, 10, 1954. <https://doi.org/10.3390/rs13101954> (2021).

NASA JPL. NASADEM Merged DEM Global 1 arc second V001 [Data set]. NASA EOSDIS Land Processes DAAC. Accessed 2020-12-30 from [doi:10.5067/MEaSUREs/NASADEM/NASADEM_HGT.001](https://doi.org/10.5067/MEaSUREs/NASADEM/NASADEM_HGT.001) (2020).

Nasirzadehdizaji, R., Sanli, F. B., Abdikan, S., Cakir, Z., Sekertekin, A. & Ustuner, M. Sensitivity analysis of multi-temporal Sentinel-1 SAR parameters to crop height and canopy coverage. *Applied Sciences.* **9** (2019).
